# Risk factors of mortality among children under age five in Awi Zone, northwest Ethiopia

**DOI:** 10.1371/journal.pone.0275659

**Published:** 2022-10-05

**Authors:** Yenew Alemu, Habtamu Dessie, Melak Birara

**Affiliations:** 1 Department of Statistics, College of Natural and Computational Science, Injibara University, Injibara, Ethiopia; 2 Department of Physics, College of Natural and Computational Science, Injibara University, Injibara, Ethiopia; Debre Tabor University, ETHIOPIA

## Abstract

**Background:**

Globally, under-five mortality rates have dropped, but in Ethiopia, the under-five mortality rate is still high. In Amhara region, the death of children under the age of five is still a public health problem. This study assessed the risk factors of mortality among children under age five in Awi Zone.

**Method:**

A community-based cross-sectional study was conducted from December 1, 2020, up to April 30, 2021. Data entry and analysis were conducted using SPSS version 26 and Stata version 16, respectively. A zero-inflated Poisson regression model was fitted to identify the risk factors of under-five mortality.

**Result:**

Out of the 1,340 mothers in the Awi zone, 11.9% of women lost at least one child. Single births (IRR = 0.598, 95% CI: 0.395, 0.906), fathers whose level of education is secondary or above(IRR = 0.223, 95% CI: 0.064, 0.782), mothers who completed their secondary and above education level(IRR = 0.116, 95% CI: 0.014, 0.971), mothers who have birth interval greater than 24 months (IRR = 0.619,95% CI: 0.417, 0.917), 8 and above family size the households (IRR = 0.543, 95% CI: 0.302, 0.976), 31 and above mother age groups (IRR = 0.296, 95% CI: 0.093, 0.943), medium households of mothers (IRR = 0.540, 95% CI: 0.316, 0.920), working mothers (IRR = 1.691, 95% CI: 1.040, 2.748) and mothers who had not antenatal visits during pregnancy (IRR = 2.060, 95% CI: 1.259, 3.371) were significant factors of under-five mortality.

**Conclusion:**

Mother’s age group, preceding birth interval, family size, wealth index, duration of pregnancy, antenatal visits during pregnancy, types of birth, mother’s education level, husband’s education level, and place of delivery were significant factors of under-five mortality in Awi zone. So, Awi zone public health institute, Awi zone children’s and youth office, and other relevant bodies should work to reduce under-five mortality by focusing on child mortality issues.

## Introduction

Under five mortality is the mortality of children under the age of five. Globally, in 2016, 5.6 million children under the age of 5 died [1]. In 2016, the global under-five mortality rate was 41 deaths per 1,000 live births and in low-income countries was 73 deaths per 1000 live births [2].

In sub-Saharan African countries, under-five mortality is higher when compared to other countries and eight times higher than the WHO European Region [3]. Similarly, sub-Saharan Africa’s under-five mortality rate remains the highest in the world. In 2019, one in 13 children in the region died before their fifth birthday. This is 15 times higher than the risk for babies born in high-income countries [4]. The under-five mortality rate is 67 deaths per 1,000 children. This means that one in 15 children in Ethiopia dies before the age of five [5].

Under-5 mortality rates for the 5 years before the survey is 67 deaths per 1,000 live births. In other words, in Ethiopia 1 of every 15 children dies before reaching the fifth birthday [5]. The current levels of child mortality in Ethiopia are higher than the target of the minimum Millennium Development Goals, which are 88 deaths per 1000 live births. In Sub-Saharan Africa, one in nine children dies before age five, more than 16 times the average for developed regions. One in 17 Ethiopian children dies before the first birthday and one in 11 Ethiopian children dies before the fifth birthday [6].

Child mortality in Ethiopia is one of the highest in the world and one of the country’s most pressing challenges. Factors such as low level of mother’s education, unsafe drinking water and sanitation, low family income, birth interval, short breastfeeding time, lack of a place of birth delivery, and periodic famine continue to put children at risk [7]. Furthermore, numerous studies have been conducted in Ethiopia and around the world reported that under-five mortality is influenced by family size [8,9], preceding birth interval [10–13], mothers occupation [14,15],type of birth[8,10,11,13,16,17], father education level [8,17], antenatal visits during pregnancy [7,17],duration of pregnancy [18], wealth index [19,20], place of delivery [7,8] and mother age group [9,17,21,22].

Under-five mortality is an indicator of population health and a measure of global health inequalities. Worldwide, the under-five mortality rate declined by 59 percent, from 93 deaths per 1,000 live births in 1990 to 38 in 2019. Despite the global growth in reducing child mortality over the past few decades, an estimated 5.2 million children under age five died in 2019, more than half of those deaths occurred in sub-Saharan Africa. In 2019, 49 percent of deaths under the age of five occurred in Nigeria, India, Pakistan, the Democratic Republic of the Congo, and Ethiopia alone. Ethiopia has the highest mortality rate in the world in less than five years [23].

In Amhara region, the death of children under the age of five is still a public health problem. The under-five mortality rate is highest in Amhara (85 deaths per 1,000 live births [5]. Under-five death is a declining figure over the last decades. However, the rate is still very high and requires a contribution to minimize death. Therefore, this study examines the main risk factors for under-five mortality in the Awi zone such as mother’s education level, economic status of the household, mother’s age at birth, length of the previous birth interval, and duration of pregnancy, etc.

Most of the researchers used binary logistic regression for under-five mortality. The dependent variable is dichotomized to be either “1” (death) or “0” (alive). For multiple child deaths, binary logistic regression model is not appropriate to use because binary logistic regression model does not provide sufficient information for studying the pattern of multiple child deaths [24]. In addition, most researchers have focused on number of under-five mortality per households, not mothers. Therefore, we compared different count regression models and discussed how women could enhance their understanding of the risk factors for under-five deaths.

Even if, little studies were conducted in the Awi zone regarded healthcare-seeking behaviors under five children, previous studies were not community-based and risk factor-specific. On the other hand, the patterns and causes of under-five mortality have not been well investigated in the Awi zone [25]. Therefore, the aim of this study was to assess the risk factors of under-five mortality in the Awi Zone, North West Ethiopia. This study might help program planners and implementers to design interventions pertaining to healthcare-seeking behavior promotion, to treat the disease of under-five children and this study may use as a baseline for researchers.

## Materials and methods

### Study area and setting

The study was conducted in Awi zone, Amhara region, North West Ethiopia from December 1, 2020, up to April 30, 2021. Awi zone is one of ten zones in Amhara region of Ethiopia. Awi zone is named for the Awi sub-group of the Agaw people, some of whom live in this zone. The administrative center of Awi zone is Injibara. This zone includes three town administration and nine rural woredas. Awi zone is bordered by Benishangul-Gumuz region to the west, North Gondar zone to the north, and West Gojam to the east. Topographically, Awi Zone is relatively flat and fertile, with elevations varying from 1,800 to 3,100 meters above sea level, with an average elevation of 2,300 meters. This zone covers an area of 9,148.43 square km. The zone is 114 km from Bahir Dar and 449 km away from Addis Ababa, the capital city of Ethiopia. Based on the 2007 Census conducted by the Central Statistical Agency of Ethiopia (CSA), this Zone has a total population of 982,942, of whom 491,865 are men and 491,077 are women [26]. Data were collected through face-to-face interviews. Women were interviewed to get information about the history of the children they gave birth to, and a total of 1340 women were considered for this study.

**Study design:** A community-based cross-sectional study was applied.

**Targeted population:** all women who had birth to at least one child residing in the Awi zone.

**Source population:** all women who gave birth in two town administrations and three districts were the source of the population.

**Study subject:** women who gave birth selected from two city administrations and three districts were the study subject.

### Ethics statement

This study was carried out in strict accordance with the recommendations in the guidance for Injibara university research institute. The procedure was approved by the Committee on the Ethics of natural and computational science research and community service (Reference number: 57/13). In addition, the written consent for asking for each participant had been obtained from the administration office of Chagini, Dangla, guagusa shikudad, Banja, and ankesha guagusa district. Verbal informed consent was also obtained from each study participant after being given an explanation of the purpose and objective of the study.

### Sample size determination

To calculate sample size, the standard deviation of the population must be considered [27]. Hence, the main outcome variable in this analysis was the number of under-five deaths experienced by individual mothers; it is better to take the standard deviation to estimate the sample size. 30 representative participants from the population of interest is a reasonable minimum recommendation for a pilot study where the purpose is a preliminary survey. The pilot survey (of 31 observations to estimate representative maximum sample size) was preferable for this study [28].

The sample size for collecting data for this study was determined by using the formula of simple random sampling [27]. The formula to estimate sample size is given as follows: $n=\frac{n_{0}}{1+\frac{n_{0}}{N}}$

$$Where,\;n_{0}=\frac{{\left(Z_{\alpha /2}\right)}^{2}s^{2}}{d^{2}}$$

n_0_ = initial sample size, if $\frac{n_{0}}{N}<5\%$, approximately n_o_ = n

S = standard deviation which was calculated from pilot survey (s^2^ = 0.209).

d = desired degree of precision.

Z_α/2_ = Z Value for the 95% level of confidence is (*z*_*α*/2_ = Critical value = 1.96)

N = total population size in the study area (in this case total number of households). The total number of households in the five selected district (Ankesha guagusa, Gugusa shikudad, Chagini town administration, Dangila town administration and Banja) which is 19,469. The total number of household for five selected district is obtained from each woreda.

n = desired the number of sample size.

The desired sample size for the study is as follows:

$$n_{0}=\frac{{\left(Z_{\alpha /2}\right)}^{2}s^{2}}{d^{2}}=\frac{{\left(1.96\right)}^{2}x\;0.209}{{\left(0.03\right)}^{2}}=\frac{0.8028944}{0.0009}=893(\mathrm{rounding}\;\mathrm{up})$$

Since $\frac{n_{0}}{N}=\frac{893}{19,469}=0.0458687898$, is less than 5%, that means n = n_0_ = 893

Design effect has two primary uses, in sample size estimation and in appraising the efficiency of more complex plans [27]. The design effect is kept as low as possible in order for the results to be usably reliable. Unless previous surveys have been conducted or similar ones in other countries so that proxy estimates of design effect can be utilized, a default value of 1.5 to 2.0 for design effect is typically used by the sampling practitioner in the formula for calculating the sample size. By minimizing or controlling the design effect as much as possible the researcher takes 1.5 for design effect. Finally: n_f_ = n*1.5 = 893*1.5 = **1340** (rounding up).

### Sampling technique

**Fig 1:**
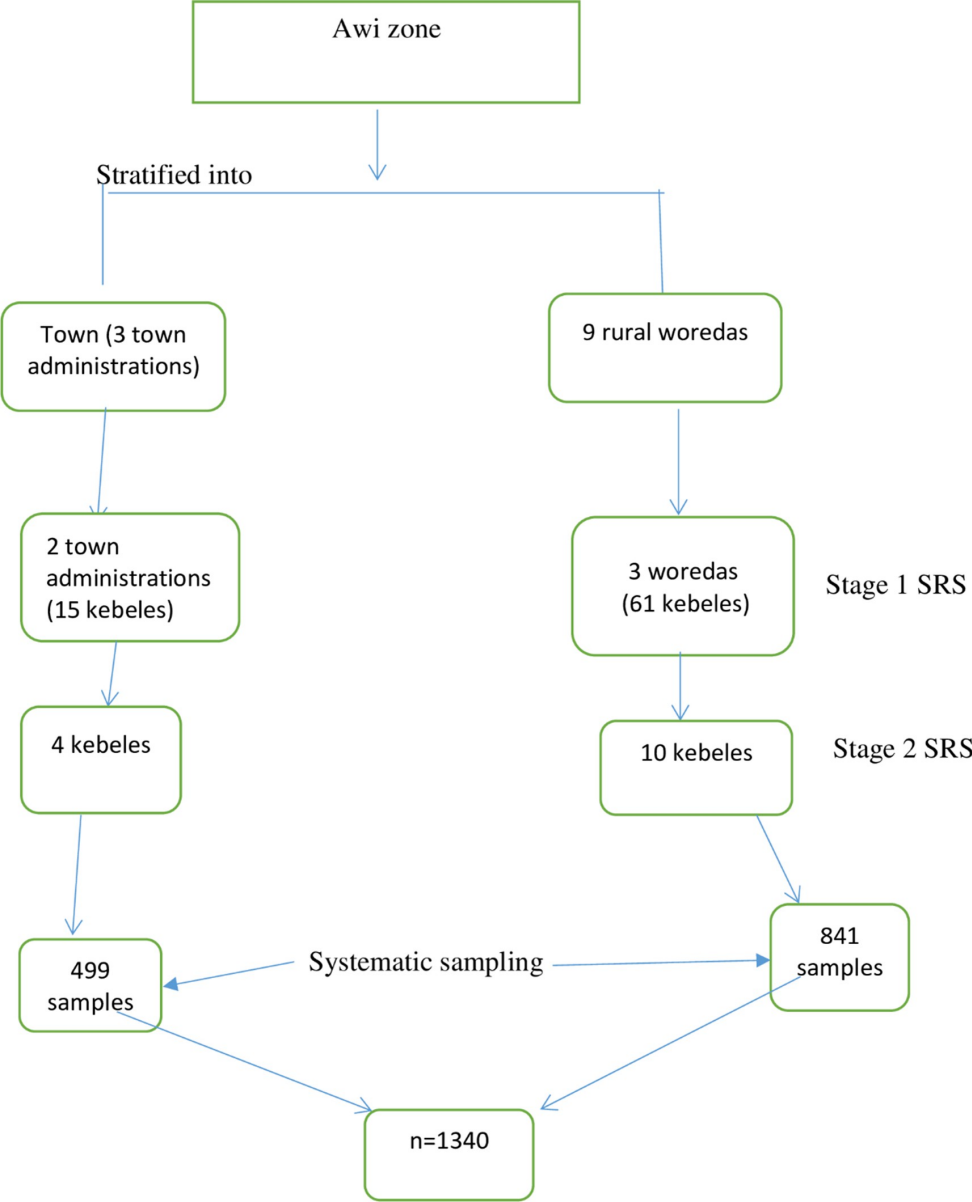
Sampling procedures and techniques.

A multi-stage stratified sampling technique was applied for this study. The detailed sampling procedure and techniques are given below in Fig 1. To select sample households from each kebele, systematic sampling technique was used with the interval of K [27]. The household lists were obtained from each kebele. The starting household for each selected kebele was selected using lottery method from the list. The number of households living in the area was recorded from each kebele.

### Inclusion and exclusion criteria

All women who had birth to at least one child residing in the Awi zone included. On the other hand, barren women, displaced mothers, women with mental illness, and hearing loss are excluded.

### Variables of the study

#### Response variable:

The response variable for this study was the number of deaths of under-five children per mother.

#### Explanatory variables:

The explanatory variables were mother’s age, types of birth, mother’s education level, preceding birth interval, source of water supply, place of delivery, antenatal visits during pregnancy, duration of pregnancy, age of mother at first birth, mother occupation, household income, marital status, availability of toilet facility, religion, types of cooking materials, place of residence, family size, breastfeeding status and education level of the father. Coding and categories of all explanatory factors are presented in Table 1.

**Table 1:** Coding and categories of all explanatory factors.

No.	Variable’s name	Categories and coding
1	Mother’s level of education	0 = No education 1 = Primary 2 = Secondary and above
2	Mothers occupation	0 = No 1 = Yes
3	Source of drinking water	0 = protected water 1 = unprotected water
4	Availability of toilet facility	0 = have toilet facility 1 = not toilet facility
5	Types of cooking material	0 = electric 1 = others
6	Place of residence	0 = urban 1 = rural
7	Preceding birth interval	0 = ≤ 24 months 1 = > 24 months
8	Wealth index	0 = poor 1 = medium 2 = rich
9	Mothers age at the first birth	0 = < 20 1 = ≥ 20
10	Religion	0 = orthodox 1 = Muslim 2 = others
11	Place of delivery	0 = home 1 = public sector 2 = private sector
12	Husband education level	0 = No education 1 = Primary 2 = Secondary and above
13	Types of birth	0 = single 1 = multiple
14	Marital status	0 = married 1 = others
15	Duration of Pregnancy	0 = < 9 months 1 = ≥ 9 months
16	Antenatal visits during pregnancy	0 = yes 1 = no
17	Mother age group	0 = below 20 1 = 20–30 2 = 31 and above
18	Breastfeeding status	0 = yes 1 = no
19	Family size	0 = ≤ 4 1 = 5–7 2 = ≥ 8

### Statistical analysis

The data were entered in SPSS software version 26 and then exported to Stata software version 16 and analyzed by using Stata software. In this study, the interest of variable (the number of deaths of under-five children per mother) is the count variable. When the dependent variable count, it is advisable to use non-linear models based on non-normal distribution. So count regression model (Poisson, NB, ZIP, and ZINB) is appropriate for this study.

Poisson regression model is a popular and simple regression model for count data. It assumes a Poisson distribution, characterized by a positive skewed and a variance equals the mean [29]. Negative binomial regression model is used when count data are over-dispersed (i.e when the variance exceeds the mean) [30]. A value of the deviance greatly in excess of degree of freedom suggests that the model is over-dispersed due to missing variables and/or non-Poisson form. Thus when deviance divided by degrees of freedom is significantly larger than 1, over-dispersion is indicated. Likewise, the Pearson chi-square statistic, defined by is an approximately chi-squared random variable with mean degree of freedom for a valid Poisson model. If the Pearson chi-square statistic is significantly larger than 1, over-dispersion is also indicated [31]. The degree of freedom is n-p. Where, n = number of observations and p = number of parameters.

The zero-inflated negative binomial regression model is one of the methods used in the over-dispersion and an excess of zeros [32]. ZINB approaches ZIP as α (over-dispersion parameter) = 0. If α = 0, NB regression model will reduce to Poisson regression model.

But based on this study, ZIP is the most appropriate model as compared to other count regression models because it has minimum AIC and maximum log-likelihood. The ZIP regression model is [33].


$$P(y_{i})=\left\{ \begin{array}{l} \omega_{i}+(1-\omega_{i})e^{-\mu_{i}},\;y_{i}=0\\ \;(1-\omega_{i})\frac{e^{-\mu i}{\mu_{i}}^{y_{i}}}{y_{i}!},\;y_{i}=1,2,\ldots ,\;0\leq \omega_{i}\leq 1 \end{array}\right.$$

The Newton-Raphson method can be used to estimate the parameter of the ZIP model.

### The goodness of fit test

The Vuong test is used to compared a non-nested model [34] whereas AIC and BIC are used for model selection of the goodness criteria. The model with the highest log-likelihood and smallest value of AIC [35] and BIC [36] is preferable. The likelihood ratio test is also a test of the overall model.

## Results and discussion

### Results

#### Descriptive statistics

Out of the 1,340 mothers in the Awi zone, 1180 (88.1%) of them never faced any child death, while the remaining 11.9% lost at least one child. Table 2 shows that 11.5% of deaths occur with uneducated mothers, while the percentage of child deaths was highly attributed to poor women (9.6%). Besides, the percent of child deaths occurred among mothers who did not receive any antenatal (9.8%). The frequency and percentage of other variables are presented in Table 2.

**Table 2:** Percentage and frequency of number of deaths of under-age five children per mother by explanatory variables.

Explanatory variables	Categories		Number of deaths of under-age five children per mother		Total
			0	≥1	
Mother age group	< 20	Freq.	22	4	26
		%	1.6	0.3	1.9
	20–30	Freq.	409	33	442
		%	30.5	2.5	33
	≥31	Freq.	749	123	872
		%	55.9	9.2	65.1
Source of drinking water	protected water	Freq.	909	94	1003
		%	67.8	7	74.8
	Unprotected water	Freq.	271	66	337
		%	20.2	5	25.2
Availability of toilet facility	Have toilet facility	Freq.	735	85	820
		%	54.9	6.3	61.2
	Not toilet facility	Freq.	445	75	520
		%	33.2	5.6	38.8
Types of cooking material	electric	Freq.	197	18	215
		%	14.7	1.3	16
	others	Freq.	983	142	1125
		%	73.4	10.6	84
Breastfeeding status	yes	Freq.	1166	125	1291
		%	87	9.3	96.3
	no	Freq.	14	35	49
		%	1	2.7	3.7
place residence	urban	Freq.	466	33	499
		%	34.8	2.4	37.2
	rural	Freq.	714	127	841
		%	53.3	9.5	62.8
family size	≤4	Freq.	465	20	485
		%	34.7	1.5	36.2
	5–7	Freq.	517	87	604
		%	38.6.	6.5	45.1
	≥8	Freq.	198	53	251
		%	14.8	3.9	18.7
preceding birth interval	≤ 24 months	Freq.	68	120	188
		%	5.1	8.9	14
	>24 months	Freq.	1112	40	1174
		%	83	3	86
respondent occupation	no	Freq.	789	128	917
		%	58.9	9.5	68.4
	yes	Freq.	391	32	423
		%	29.2	2.4	31.6
Wealth index	poor	Freq.	208	129	337
		%	15.5	9.6	25.1
	medium	Freq.	878	27	905
		%	65.5	2.1	67.6
	rich	Freq.	94	4	98
		%	7	0.3	7.3
mother age at the first birth	< 20	Freq.	218	126	344
		%	16.3	9.4	25.7
	≥20	Freq.	962	34	996
		%	71.8	2.5	74.3
religion	orthodox	Freq.	1088	145	1233
		%	81.2	10.8	92
	Muslim	Freq.	68	14	82
		%	5.1	1	6.1
	others	Freq.	24	1	25
		%	1.8	0.1	1.9
place of delivery	home	Freq.	106	138	244
		%	7.9	10.3	18.2
	Public sector	Freq.	987	20	1007
		%	73.7	1.5	75.2
	Private sector	Freq.	87	2	89
		%	6.5	0.1	6.6
respondent education level	No education	Freq.	579	154	733
		%	43.2	11.5	54.7
	primary	Freq.	220	5	225
		%	16.4	0.4	16.8
	Secondary and above	Freq.	381	1	382
		%	28.4	0.1	28.5
Husband education level	No education	Freq.	497	149	646
		%	37.1	11.1	48.2
	primary	Freq.	267	8	275
		%	19.9	0.7	20.6
	Secondary and above	Freq.	416	3	419
		%	31	0.2	31.2
Type of birth	multiple	Freq.	2	19	21
		%	0.1	1.5	1.6
	single	Freq.	1178	141	1319
		%	87.9	10.5	98.4
marital status	Currently married	Freq.	1154	153	1307
		%	86.1	11.4	97.5
	others	Freq.	26	7	33
		%	1.9	0.6	2.5
pregnant length	< 9 months	Freq.	64	77	141
		%	4.8	5.7	10.5
	≥ 9 months	Freq.	1116	83	1199
		%	83.3	6.2	89.5
Antenatal visit	yes	Freq.	1030	28	1058
		%	76.9	2.1	79
	no	Freq.	150	132	282
		%	11.2	9.8	21

**Fig 2:**
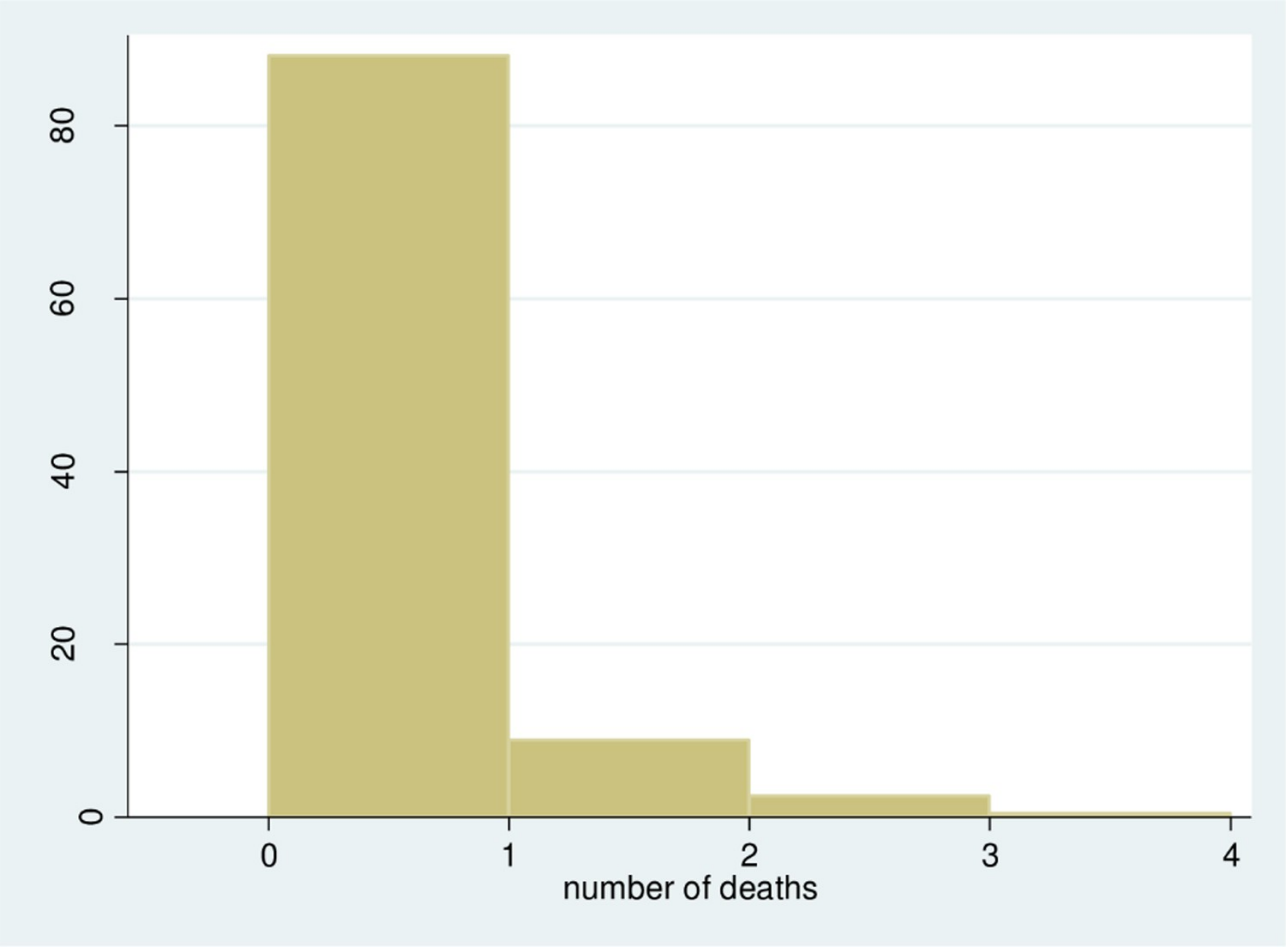
Hostogram of the number of under-five deaths per mother.

Fig 2 indicates that the zero results are large in number. Histograms were initially high at the beginning. But, the number of mothers under the age of five mortality is relatively low.

This implies the distribution is non-normal or positively skewed.

Based on the data, the Deviance statistics (176.985) and Pearson Chi-square statistic (248.909) divided by their corresponding degrees of freedom (1313) are less than one, respectively. This implies there is no over-dispersion. So Poisson model is the most appropriate model as compared to the negative binomial model. In addition, the fact that the Likelihood ratio test of α statistic, LRT_α_ = -2(Log-likelihood of Poisson–Log-likelihood of NB) = -2(-262.227 + 262.227) = 0. The value of the likelihood-ratio test of dispersion parameter alpha was 0 with p- value of 0.499 which demonstrated that there is an no over dispersion in our data because the p-value is greater than the level of significance (0.05) that means failing to reject the null hypothesis (α = 0). P-value of LRTα > level of significance implies Poisson regression is preferred as compared to NB. We cannot proceed with the negative binomial and zero-inflated negative binomial regression models due to no over-dispersion. But we used ZIP regression model because it has an excess zeros (Table 3).

**Table 3:** Model comparisons using Vuong test for non-nested.

Model	Vuong Statistics(V)	P-value	Preferred model
ZIP vs Poisson		0.003	ZIP

For non-nested models, ZIP versus Poisson regression models was identified using the Vuong test statistic. This implies ZIP is the most appropriate model (Table 3).

Based on Table 4, ZINB is the most preferred as compared to NB because it has maximum Log-likelihood and minimum AIC. Table 4 shows that ZIP is the most appropriate model as compared to others. Because it has maximum log-likelihood and minimum AIC. BIC is unfortunately high, using the default BIC calculation is not probably fine. There is an issue, but most researchers would ignore BIC.

**Table 4:** Summary of fit statistics.

Model	Log-likelihood	AIC	BIC
Poisson	-262.227	578.454	718.866
NB	-262.227	578.454	718.866
ZIP	-249.843	569.687	751.702
ZINB	-249.843	571.687	758.902

**Fig 3:**
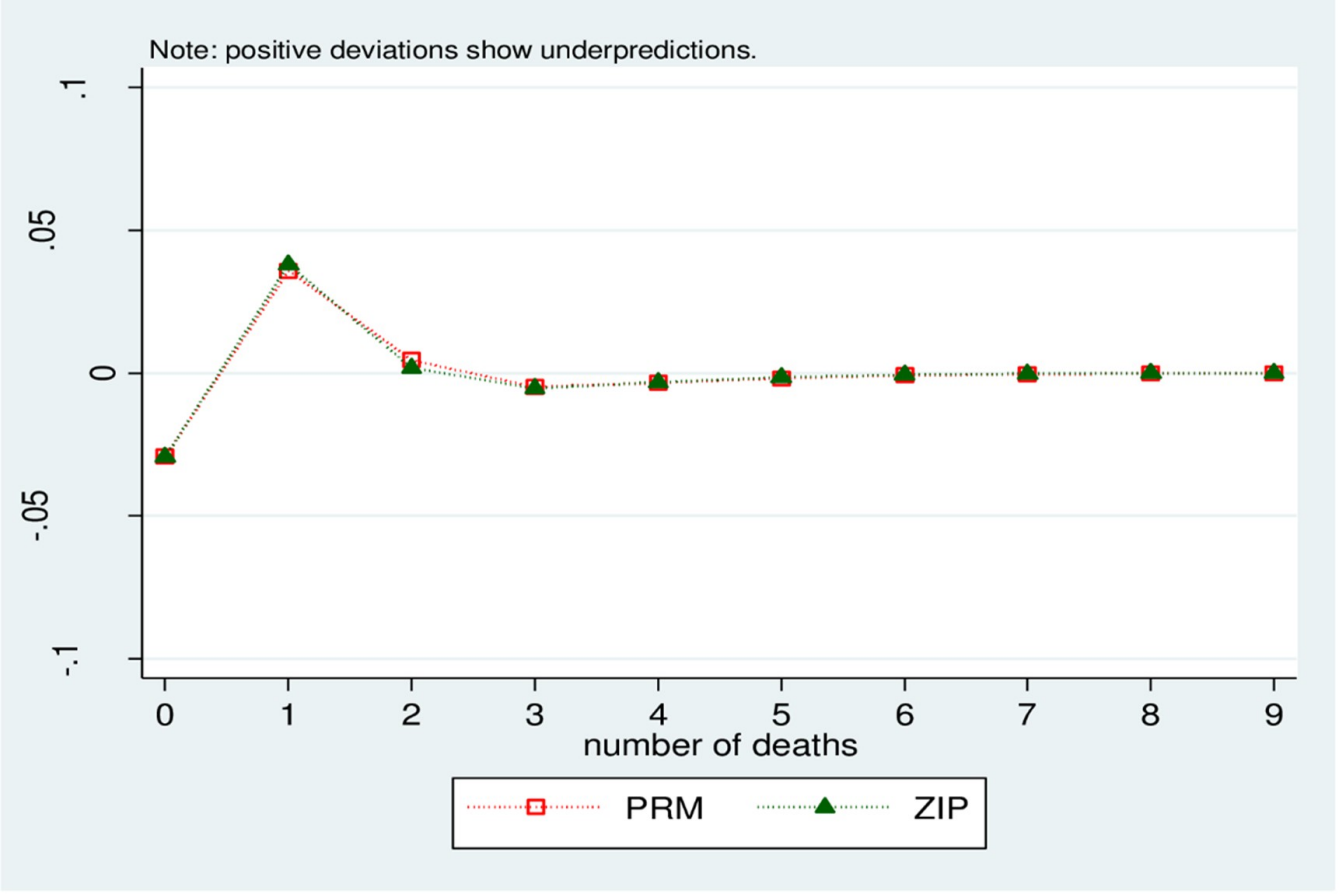
Difference between observed and predicted proportions for number of under five deaths per mothers.

The model was compared by using an observed versus predicted probability plot. The residual plot in Fig 3 confirms that the ZIP model is preferred and it is the most appropriate model among the count models because almost all ZIP points pass 0 and form a straight line after a period of fewer than five years of mortality.

### The risk factors of mortality under the age of children in Awi Zone

This study was to investigate the risk factors of under-five deaths in Awi zone. The ZIP regression model parameter estimation for non-zero groups are presented in Table 5.

**Table 5:** ZIP regression model parameter estimation for non-zero groups.

Explanatory variables	IRR	Std. Err.	z	P>|z|	95% CI
Source of drinking water, protected (ref.)					
Unprotected	1.044	0.198	0.23	0.821	0.720, 1.513
Types of toilet facility, comfortable (ref.)					
Uncomfortable	0.983	0.175	-0.09	0.924	0.693, 1.394
Breastfeeding status, yes(ref)					
No	1.203	0.220	1.01	0.312	0.841, 1.720
Place of residence, urban (ref.)					
Rural	0.911	0.260	-0.33	0.745	0.521, 1.593
Types of cooking material, electric(ref.)					
Others	0.662	0.212	-1.29	0.197	0.353, 1.239
Marital status, currently married (ref.)					
Unmarried	1.671	0.612	1.40	0.161	0.815, 3.426
Religion, orthodox (ref.)					
Muslim	1.485	0.508	1.16	0.248	0.759, 2.905
Others	0.582	0.602	-0.52	0.601	0.077, 4.422
Family size, (≤4)(ref.)					
5–7	1.022	0.279	0.08	0.937	0.598, 1.746
8 and above	0.543	0.162	-2.04	0.041*	0.302, 0.976
Preceding birth order, (≤24 months)(ref.)					
>24 months	0.619	0.124	-2.39	0.017*	0.417, 0.917
Mother age groups, <20 (ref.)					
20–30	0.618	0.362	-0.82	0.411	0.196, 1.946
31 and above	0.296	0.175	-2.06	0.039*	0.093, 0.943
Mothers occupation, no (ref.)					
Yes	1.691	0.419	2.12	0.034*	1.040, 2.748
Wealth index, poor (ref.)					
Medium	0.540	0.147	-2.27	0.023*	0.316,0.920
Rich	0.328	0.196	-1.87	0.062	0.102, 1.040
Mother age at the first birth, <20 (ref.)					
> = 20	0.689	0.159	-1.62	0.106	0.437, 1.083
Place of delivery, home (ref.)					
public sector	0.641	0.178	-1.60	0.110	0.372, 1.106
Private sector	0.493	0.405	-0.86	0.390	0.098, 2.469
Mother’s education level, no education (ref.)					
Primary	0.420	0.204	-1.79	0.074	0.162, 1.088
Secondary and above	0.116	0.126	-1.99	0.047*	0.014, 0.971
Husband education level, no education (ref.)					
Primary	0.473	0.186	-1.90	0.057	0.219, 1.022
Secondary and above	0.223	0.143	-2.34	0.019*	0.064, 0.782
Types of birth, multiple (ref.)					
Single	0.598	0.127	-2.42	0.015*	0.395, 0 .906
Duration of pregnancy, < 9 months (ref.)					
> = 9 months	0.761	0.127	-1.64	0.101	0.549, 1.055
Antenatal visits during pregnancy, yes (ref.)					
No	2.060	0.518	2.88	0.004*	1.259, 3.371
_cons	1.421	1.032	0.48	0.629	0.342, 5.898
Ln(total children ever born)	1(offset)				

ref. = reference category of the variable.

* Significant at 0.05 level of significance.

Based on Table 5, the family size was a significant factor in under-five deaths. The incidence rate of under-five children death for a family size of eight and above was 0.543(IRR = 0.543, 95% CI:0.302,0.976) times less likely as compared to the family size of less than five while holding all other variables in the model constant. The risk of under-five death decreased as the family size of the household increased, meaning that children in a larger household had a better chance of surviving to see their fifth birthday.

Table 5 showed that the preceding birth interval had statistically significant on under-five deaths. The expected number of under-five deaths per mother for preceding birth interval of above 24 months was 38.1% (IRR = 0.619,95%CI: 0.417, 0.917) less than the expected number of under-five deaths for preceding birth interval of less than or equal to 24 months controlling other variables in the model. In addition, the expected number of under-five deaths for working mothers was 1.691 (IRR = 1.691, 95%CI: 1.040, 2.748) times more likely than not working mothers (housewives) while holding all other variables in the model constant.

According to the findings of this study, antenatal visits during pregnancy have a significant influence on the number of under-five deaths per mother. The incidence rate of non-zero under-five death among mothers who had not antenatal checks during the pregnancy was 2.06 (IRR = 2.060, 95%CI: 1.259, 3.371) times more likely as compared with mothers who have received any antenatal check.

When we look at the wealth index of the household, the expected number of under-five deaths for women in the medium households was 0.540 (IRR = 0.540, 95%CI: 0.316, 0.920) times less likely than the expected number of under-five deaths for women in the poor households, while holding all other variables in the model constant.

The finding of this study also revealed that mothers’ level of education had a significant factor in the number of under-five deaths. Mothers were less likely to have a positive count of under-five deaths of their children with the secondary and above levels of education. The expected number of under-five deaths for mothers who attend secondary and above education was about 0.116 (IRR = 0.116, 95%CI: 0.014, 0.971) times less than that of the expected number of under-five deaths for mothers who did not attend any formal education, controlling for the other variables in the model. Likewise, a husband of women was less likely to have a risk of under-five deaths of his children with the secondary and above level of education. The incidence rate of non-zero under-five death for husbands of women with secondary education and above was 0.223 (IRR = 0.223, 95%CI: 0.064, 0.782) times lower compared to fathers with no formal education.

On the other hand, women who have experienced under-five deaths were less likely for single birth as compared to the multiple births in Awi zone. The risk of under-five death in single birth was 0.598 (IRR = 0.598, 95%CI: 0.395, 0.906) times less than compared with that of multiple births holding all other variables in the model constant. Moreover, the expected number of under-five deaths for mothers age 31 and above was 0.296(IRR = 0.296, 95%CI: 0.093,0.943) times less likely as compared to mother age group of below 20 holding all other variables in the model constant.

The ZIP regression model parameter estimation for zero groups are presented in Table 6.

**Table 6:** ZIP regression model parameter estimation for zero groups.

Explanatory variables	OR	Std. Err.	z	P>|z|	95% CI
Place of delivery, home (ref.)					
public sector	6.725	2.155	3.12	0.002*	2.501, 10.950
Private sector	7.562	3.15	2.40	0.016*	1.386, 13.738
Wealth index, poor (ref.)					
Medium	3.109	1.847	1.68	0.092	-0.510, 6.729
Rich	7.690	3.667	2.10	0.036*	0.502, 14.877
Mother’s occupation, no (ref.)					
Yes	-4.203	1.895	-2.22	0.027*	-7.918, -0.488
Mother age at the first birth, <20 (ref.)					
> = 20	1.949	1.452	1.34	0.180	-0.897, 4.795
Duration of pregnancy, <9months (ref.)					
> = 9 months	4.482	1.984	2.26	0.024*	0.593, 8.370
Cons	-10.746	2.300	-3.58	0.000	-16.626, -4.867

ref. = reference category of the variable.

* Significant at 0.05 level of significance.

As shown in Table 6, the odds ratio of zero under-five death for children born in the private health facility and public sector were 7.562 and 6.725 times more than that of children born at home respectively, holding all other variables in the model constant. In addition, the odds ratio of zero under-five death for the duration of pregnancy 9 months and above mothers was 4.482 times more than that of the duration of pregnancy of mothers below 9 months.

The finding of this study also revealed that the odds of the number of children under the age of five death becomes zero with children born from mothers who work was 4.203 times lower than that of mothers without work (housewife). Moreover, the result of this study for the inflated group as displayed in Table 6, showed that the wealth index was found to be a statistically significant factor for under-five mortality in the Awi zone. By keeping other variables held constant in the model, the estimated odds that the number of under-five death becomes zero among mothers belong to the rich wealth index of households was 7.690 times more than mothers belongs to the poor wealth index of households in the Awi zone.

### Discussion

This study was carried out to identify the risk factors of under-five deaths in Awi zone. The total number of women from Awi zone included in the present study was 1,340 among which 11.9% experienced under-five deaths due to different factors. The study revealed that family size, mother age group, preceding birth interval, wealth index, duration of pregnancy, antenatal visits during pregnancy, types of birth, mother’s education level, husband education level and place of delivery were risk factors for U5CM in Awi zone.

Family size was a significant factor on under five deaths. The odds ratio of under five children death for family size of eight and above was less likely as compared to family size of less than five while holding all other variables in the model constant. The risk of under-five death decreased as family size of the household increased, meaning that children in a larger household had a better chance of surviving to see their fifth birthday. This is because a mother who has more than one child has the experience of seeking health care services to keep her child healthy. This finding is consistent with other studies [8,9].

The finding revealed that under five mortality decreased with an increase in the length of the preceding birth interval. Mothers who gave birth with preceding birth intervals of shorter than 24 months was a significant association with under-five deaths. Short birth interval makes worse for maternal and under five children malnutrition. This can pose a serious risk to maternal nutrition and child development, which in turn increases the risk of under-five mortality due to malnutrition. This study is confirm with findings from previous studies [10–13].

The result showed that mother’s occupation was found to be statistically significant with under-five deaths. Being employed mothers or children from working mothers have a higher risk of death than those from non-working mothers. This is because educated mothers have better knowledge and experience in basic health services, including immunizations, disease treatment, preventive care, hygiene, and nutrition. This finding is consistent with the previous studies [15] but inconsistent with others study [14].

According to the result of this study, Types of birth was found to be statistically significant factor for under-five death. Child death with multiple births is higher relative to single. Multiple births have a lower weight due to diet intake competition. This finding is consistent with those of previous studies [8,10,11,13,16,17].

The finding revealed that husband of women or father of a child was found to be statistically significant factor for under-five death. The result indicates that the risk of under-five death was low for husband of women who has secondary and above education level as compared to father of a child who have no education level keeping other variables held constant in the model. The possible explanation is the higher the level of education of fathers, the higher the chances of children surviving. Increasing paternity education increases mothers’ awareness of children’s health and hygiene. This finding is consistent with [9,17].

According to the findings of this study, antenatal visits during the pregnancy has a significant influence on under-five deaths per mother. The odds ratio of under-five death among mothers who had not antenatal visits during the pregnancy was more likely as compared with mothers who have received any antenatal visit. This is because mothers who do not visit prenatal care may have problems during pregnancy, and fetal health may be high in risk. This result is supported by the previous research [7,17].

The current study revealed that duration of pregnancy for women was a significant factor on the age of under five children deaths. Children born before nine months were more likely to die as compared to children born at nine and above month pregnancy period. The reason is due to the child may be infected by pneumonia or other diseases. This study is stable with [18].

In addition, wealth index of the households also another important significant factors for under five deaths. The risk of under-five deaths per mothers in the medium and rich households was less likely as compared to mothers in the poor households. Wealth conditions determine the safety of children. Medium and rich families are more likely to take their children to the health facility of their choice when children are sick. In addition, they can provide better nutrition, shelter and health services to children. This is consistent with [19,20].

Place of delivery was a significant effect on under the age of five children deaths. Children born in public and private health institutions are at lower risk than those born at home. This may be due to established better health care and attention before and after childbirth. This has been corroborated by different studies [7,8]. Moreover, the death decreased significantly as the mother age group increased due to she had gained more experience in child rearing and older children would be better able to take care of their newborns and mothers would be more likely to care for their babies because it might be the last child birth, a finding which is unexpected and inconsistent with finding from previous studies [9,17].

In this study, mothers’ education was found to be an important predictor on under-five deaths in Awi zone. Under the age of children death decrease with increased level of mother’s education indicating that improved mothers education minimizes the number of age children under the age five deaths. This is because educated mothers have better knowledge and experience in basic health services, including immunizations, disease treatment, preventive care, hygiene, and nutrition. This result is consistent with the findings by [13,16,37,38].

## Conclusions

Among the four models considered for analyzing the data from women in the Awi zone, the ZIP regression model was found to be the most appropriate model. The study revealed that mother’s age group, preceding birth interval, wealth index, mother occupation, mother’s level of education, husband level of education, antenatal visits during pregnancy, duration of pregnancy, types of birth, and family size had a statistically significant effect on the mortality of children under the age five in Awi zone. Based on our findings we recommend that health professionals and stakeholders should educate mothers about antenatal care and the importance of mothers giving birth in health facilities and establish a daycare center around the workplace for working mothers or allow to enter by shift for working mothers to take care for their children. In addition, health professionals, mothers, fathers and family members should take proper care of twins and babies born before 9 months. As well as the Awi zone public health institute, Awi zone children and youth the office, and all concerned bodies should implement a child-centered program in each woreda and kebele. In order to reduce the mortality of children under the age of five, it is important to assign health workers in woredas and kebeles and improve women’s education.

### Strength and limitation of the study

The strength of this study was the selected women represent the study population and the selection process was well designed. The study was only interviewed surviving women except for barren women, displaced mothers, women with mental illness, and hearing loss women. Therefore, no data were available for children of women who had died.

## Supporting information

S1 Dataset (CSV)

## Abbreviations

AIC: Akaike Information Criteria
BIC: Bayesian Information Criteria
ZIP: Zero Inflated Poisson
NBRM: Negative Binomial
ZINB: Zero Inflated Negative binomial
SPSS: Statistical Product and Service Solutions
STATA: South Texas Art Therapy Association
OR: Odds Ratio
IRR: Incidence Rate Ratio
U5CM: Under-Five Child Mortality
CI: Confidence Interval
CSA: Central Statistical Agency
LRT: Likelihood Ratio Test
